# Gut Microbial Dysbiosis and Changes in Fecal Metabolic Phenotype in Precancerous Lesions of Gastric Cancer Induced With N-Methyl-N′-Nitro-N-Nitrosoguanidine, Sodium Salicylate, Ranitidine, and Irregular Diet

**DOI:** 10.3389/fphys.2021.733979

**Published:** 2021-11-04

**Authors:** Fuhao Chu, Yicong Li, Xiangmei Meng, Yuan Li, Tao Li, Mengyin Zhai, Haocheng Zheng, Tianxi Xin, Zeqi Su, Jie Lin, Ping Zhang, Xia Ding

**Affiliations:** ^1^Institute of Regulatory Science for Traditional Chinese Medicine, Beijing University of Chinese Medicine, Beijing, China; ^2^School of Traditional Chinese Medicine, Beijing University of Chinese Medicine, Beijing, China; ^3^Research Center for Spleen and Stomach Diseases of Traditional Chinese Medicine, Beijing University of Chinese Medicine, Beijing, China; ^4^Dongfang Hospital, Beijing University of Chinese Medicine, Beijing, China; ^5^Dongzhimen Hospital, Beijing University of Chinese Medicine, Beijing, China; ^6^Beijing Research Institute of Chinese Medicine, Beijing University of Chinese Medicine, Beijing, China; ^7^Wangjing Hospital, China Academy of Chinese Medical Sciences, Beijing, China

**Keywords:** precancerous lesions of gastric cancer (PLGC), gut microbial and metabolic dysbiosis, MNNG, *16S rRNA* genes sequencing, untargeted metabolomics analysis

## Abstract

**Background and Aims:** Precancerous lesions of gastric cancer (PLGC) are the most important pathological phase with increased risk of gastric cancer (GC) and encompass the key stage in which the occurrence of GC can be prevented. In this study, we found that the gut microbiome changed significantly during the process of malignant transformation from chronic gastritis to GC in N-methyl-N′-nitro-N-nitrosoguanidine (MNNG) multiple factors-induced rat model. Accumulating evidence has shown that alterations in gut microbiota and metabolism are potentially linked to chronic inflammation and cancer of the gastrointestinal tract. However, the correlation of gut microbiota and metabolites, inflammatory factors, and the potential mechanism in the formation of PLGC have not yet been revealed.

**Methods:** In this study, multiple factors including MNNG, sodium salicylate drinking, ranitidine feed, and irregular diet were used to establish a PLGC rat model. The pathological state of the gastric mucosa of rats was identified through HE staining and the main inflammatory cytokine levels in the serum were detected by the Luminex liquid suspension chip (Wayen Biotechnologies, Shanghai, China). The microbial composition and metabolites in the stool samples were tested by using *16S ribosomal RNA* (*rRNA*) gene sequencing and non-targeted metabolomics. The correlation analysis of gut microbiota and inflammatory cytokines in the serum and gut microbiota and differential metabolites in feces was performed to clarify their biological function.

**Results:** The results showed that compared to the control group, the gastric mucosa of the model rats had obvious morphological and pathological malignant changes and the serum levels of inflammatory cytokines including interleukin-1β (IL-1β), interleukin-4 (IL-4), interleukin-6 (IL-6), interleukin-10 (IL-10), interferon-γ (IFN-γ), tumor necrosis factor-α (TNF-α), and macrophage colony-stimulating factor (M-CSF) increased significantly, while the level of chemokine (C-X-C motif) ligand 1 (CXCL1) in serum reduced significantly. There were significant differences in the composition of the gut microbiota and fecal metabolic profiles between the model and control rats. Among them, *Lactobacillus* and *Bifidobacterium* increased significantly, while *Turicibacter*, *Romboutsia*, *Ruminococcaceae_UCG-014*, *Ruminococcaceae_UCG-005*, and *Ruminococcus_1* reduced significantly in the model rats compared to the control rats. The metabolites related to the lipid metabolism and peroxisome proliferator-activated receptor (PPAR) signaling pathway have also undergone significant changes. In addition, there was a significant correlation between the changes of the differential inflammatory cytokines in the serum, fecal metabolic phenotypes, and gut microbial dysbiosis in model rats.

**Conclusion:** The activation of the inflammatory response, disturbance of the gut microbiota, and changes in the fecal metabolic phenotype could be closely related to the occurrence of PLGC. This study provides a new idea to reveal the mechanism of risk factors of chronic gastritis and GC from the perspective of inflammation-immune homeostasis, gut microbiota, and metabolic function balance.

## Introduction

Gastric carcinoma (GC) is the fifth most frequently diagnosed cancer and the third leading cause of cancer-related death worldwide (Bray et al., 2018), and approximately half of the world GC cases and deaths occur in China (Nie et al., 2017). Evidence from the pathology and epidemiology studies indicated that the human model of gastric carcinogenesis evolved from the following sequential stages known as Correa cascade: chronic non-atrophic gastritis (CNAG), followed by precancerous lesions of gastric cancer (PLGC), including chronic atrophic gastritis (CAG), intestinal metaplasia (IM), and dysplasia (DYS) (Correa et al., 1975; Correa, 1992; Park and Kim, 2015), eventually worsened into gastric adenocarcinoma. PLGC is the most important pathological phase with increasing the risk of gastric cancer (Correa et al., 1990, 2010; You et al., 1993; de Vries et al., 2008; Song H. et al., 2015; Li et al., 2016). Exploring the pathogenesis of the PLGC stage could provide new strategies for preventing the occurrence of GC.

The occurrence of GC is closely related to *Helicobacter pylori* (*H. pylori*) infection, dietary habits, gastroesophageal reflux, long-term stomach inflammation, and proton pump inhibitors (PPIs) abuse (Engel et al., 2003; Carrasco and Corvalan, 2013; Palmer, 2017; Laterza et al., 2019). Long-term exposure to *H. pylori* infection is one of the main etiologic factors for the progression of PLGC and GC (Díaz et al., 2018). *Helicobacter pylori* infection could release a variety of bacterial virulence factors (VacA, CagA, etc.), which could promote gastric cell death, induce genetic and epigenetic changes in the gastric epithelial cells and the initial inflammatory response, and eventually resulting in primary tissue lesions (Valenzuela et al., 2015). More notably, as the food additives in processed meats, consumption of nitrates was related to a decreased risk of GC, while high intake of nitrites and N-nitrosodimethylamine (NDMA) resulted in an increased risk of GC (Song P. et al., 2015). In addition, drug abuse could also cause inflammation and damage to the gastric mucosa, which, in turn, increased the risk of GC. Among them, PPI abuse could inhibit the production of gastric acid and then contribute to gastric cancer pathogenesis, which could be related to the growth of excessive pathogenic microorganisms in a low-acid state in the stomach (Palmer, 2017; Laterza et al., 2019). The non-steroidal anti-inflammatory drugs (NSAIDs) are well-known cyclooxygenase (COX) inhibitors for the treatment and prevention of cancer due to the relationship between chronic inflammation and cancer (Wong, 2019). However, there are conflicting findings on the role of NSAIDs in the prevention and treatment of cancer. Long-term NSAID use is often associated with serious gastrointestinal (GI), cardiovascular, renal, and other side effects (Harirforoosh et al., 2013) and increased risk or mortality in certain types of cancer (Brasky et al., 2011; Choueiri et al., 2014). Although the report about the dietary habits and drugs abuse increasing the risk of gastritis and gastric cancer is mostly epidemiologic, the mechanisms underlying the increased risk are less well delineated.

Increasing evidence has shown that alterations in gut microbiota are potentially linked to inflammation and cancer (Schwabe and Jobin, 2013; Abreu and Peek, 2014; Garrett, 2015; Vogtmann and Goedert, 2016; Bhatt et al., 2017). The gut microbiota consists of a huge number of bacteria, which participate in the absorption of nutrients, energy metabolism, maturation of the intestinal immune system, and protection of GI mucosa from the infection of pathogens (Lozupone et al., 2012; Nicholson et al., 2012). The gut microbiota is easily influenced by a variety of factors including diet, age, antibiotics, and environmental and psychological factors (Osadchiy et al., 2019). The gut microbial dysbiosis resulted in immunological dysregulation has been associated with the pathogenesis of *H. pylori* infection, chronic inflammation, and cancer (Round and Mazmanian, 2009; Lam et al., 2017; Zitvogel et al., 2017; Wong et al., 2019). The increased risk of gastritis and GC caused by the unhealthy diet and drug abuse is related to the imbalance of gut microbiota that has not been elucidated.

N-Methyl-N′-nitro-N-nitrosoguanidine (MNNG)-induced mouse model, one of the chemical carcinogenesis models, has been widely used for the GC and development of gastric precancerous lesions (Cai et al., 2018, 2019; Xu et al., 2018; Zhang et al., 2018; Zhao et al., 2019). In this study, we modified the MNNG-induced model to combined with sodium salicylate drinking, ranitidine feed, and irregular diet (multifactor induction) that were used to simulate the main factors leading to chronic gastritis and GC such as irregular diet, gastric mucosal inflammation damage, and PPI abuse to establish PLGC rat model regarding our previous report (Yu et al., 2020).

To explore the mechanisms of the interaction of gastric inflammation, gut microbiota, and metabolic in PLGC, we used the multifactor-induced rat PLGC model and performed the serum cytokines detection, gut microbiota 16S ribosomal RNA (rRNA) sequence, and fecal non-targeted metabolomics assay.

## Materials and Methods

### Materials

N-Methyl-N′-nitro-N-nitrosoguanidine was purchased from the TCI (Shanghai) Huacheng Industrial Development Corporation Ltd. (Shanghai, China). Sodium salicylate was purchased from the Sinopharm Chemical Reagent Corporation Ltd. (Beijing, China) and prepared 2% solution with the SPF-grade animal drinking water every day. Granulated SPF-grade rat fodder containing 0.05% ranitidine was purchased from the Beijing Keao Xieli Feed Corporation Ltd. (Beijing, China). All the other chemicals used in ultra performance liquid chromatography-quadrupole-time-of-flight mass spectrometry (UPLC-Q-TOF/MS) were of MS grade and purchased from the Thermo Fisher Scientific Incorporation (Waltham, MA, United States).

### Animal Study

**FIGURE 1:**
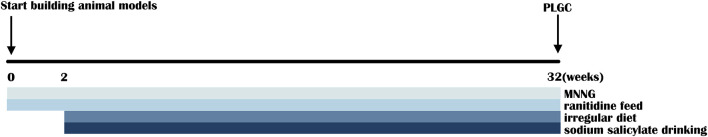
Schematic view of the establishment of multifactor-induced precancerous lesions of gastric cancer (PLGC) rat model.

A total of 18 SPF-grade Wistar rats (male, 100–130 g, 4-week-old) were purchased from the Beijing Vital River Laboratory Animal Technology Corporation Ltd. (Beijing, China). All the animal experiments were approved by the Ethics Committee of Beijing University of Chinese Medicine. Animals were received food and water under the SPF conditions with a standard 12 h light/dark cycle, a temperature of 22 ± 2°C, and relative humidity of 60 ± 5%. After 1 week of acclimatization, all the 18 rats were randomly divided into the control group and model group. The rats of the model group were free to drink 120 μg/ml of MNNG aqueous solution and feed with granular SPF-grade fodder containing 0.05% ranitidine and the rats of the control group were given with normal chow and water. Besides, on every Tuesday and Friday, abrosia and gavage of 0.05 mL/kg 2% sodium salicylate solution were given to rats of the model group and normal feeding and gavage of 0.05 ml/kg water were given to rats of the control group (Figure 1). After the 32nd week, the feces of all the rats were collected in separate cryotubes and stored at −80°C for the detection of gut microbiota and metabolites. Rats were anesthetized by intraperitoneal injection with 0.2 ml/100 g 2% pentobarbital sodium solution after fasting for 18 h. The whole stomach was removed and cut along the stomach bend. After removed the contents and washed with physiological saline, the stomach tissue was unfolded on the filter paper to observe whether mucosal damage or not takes place.

### Histopathological Analysis

The histopathological analysis of gastric mucosa tissues was performed on all the rats. The samples were embedded in paraffin and sectioned to 4 μm slides. The sections were stained by using HE dying and then observed by using light microscopy. The HE scores were evaluated by the professional pathology researchers in our experiment according to the Gastritis and Gastric Cancer Scale. Scoring criteria were as follows: (1) Multifocal aggregates of mononuclear ± polymorphonuclear leukocytes; (2) Coalescing aggregates of the inflammatory cells in submucosa ± mucosa; (3) Organizing nodules of the lymphocytes and other inflammatory cells in submucosa ± mucosa; and (4) Follicles or sheets of the inflammatory cells extending into or through muscularis ± adventitia (Rogers, 2012).

### High-Throughput Detection of Inflammatory Cytokines in Serum

The levels of inflammatory cytokines in the serum of rats in each group were detected by the Luminex liquid suspension chip (Wayen Biotechnologies, Shanghai, China). The Bio-Plex Pro Rat Cytokine 23-Plex Group I Panel System (Hercules, CA, United States) was used following the instructions of the manufacturer. Rat serum was incubated in 96-well plates embedded with microbeads for 1 h and then incubated with detection antibody for 30 min. Subsequently, streptavidin-phycoerythrin (PE) was added to each well for 10 min and values were read by using the Bio-Plex MAGPIX System (Bio-Plex) (Hercules, CA, United States).

### Gut Microbiota Analysis

Microbial DNA was extracted from the fecal samples of rats by using the E.Z.N.A.^®^ Soil DNA Kit (Omega Bio-Tek Incorporation, Norcross, GA, United States) according to the protocols of the manufacturer. The final DNA concentration and purity were determined by the NanoDrop^TM^ 2000c UV-Visible Spectrophotometer (Thermo Scientific, Wilmington, DE, United States) and DNA quality was checked by 1% agarose gel electrophoresis. The V3–V4 hypervariable regions of the bacteria *16S rRNA* gene were amplified with primers 338F (5′-ACTCCTACGGGAGGCAGCAG-3′) and 806R (5′-GGACTACHVGGGTWTCTAAT-3′) by thermocycler PCR system (GeneAmp 9700 PCR System 9700, ABI, CA, United States). PCR reactions were performed in triplicate 20 μl mixture containing 4 μl of 5 × FastPfu Buffer, 2 μl of 2.5 mM dNTPs, 0.8 μl of each primer (5 μM), 0.4 μl of FastPfu DNA Polymerase, and 10 ng of template DNA. The PCR reactions were conducted three repetitions independently by using the following program: 3 min of denaturation at 95°C, 27 cycles of 30 s at 95°C, 30 s for annealing at 55°C, 45 s for elongation at 72°C, and a final extension at 72°C for 10 min. The resulted PCR products were extracted from a 2% agarose gel and further purified by using the AxyPrep DNA Gel Extraction Kit (Axygen Biosciences, Union City, CA, United States) and quantified by using the QuantiFluor^TM^-ST (Promega Corporation, United States) according to the protocol of the manufacturer. The amplified complementary DNA (cDNA) was built by the following steps: first, the fragment was connected to the “Y” glyph connector, the self-connected fragment of the connector was removed by magnetic bead screening, then the library template was enriched by PCR amplification, and finally, sodium hydroxide degeneration was used to produce a single-stranded DNA fragment. Purified amplicons were pooled in equimolar and paired-end sequencing (2 × 300) on the Illumina MiSeq Platform (Illumina, San Diego, CA, United States) according to the standard protocols by the Majorbio Bio-Pharm Technology Corporation Ltd. (Shanghai, China). Raw FastQ files were quality filtered by trimmomatic and merged by Fast Length Adjustment of SHort reads (FLASH) with the following criteria: (i) The reads were truncated at any site receiving an average quality score < 20 over a 50-bp sliding window, (ii) Sequences whose overlap being longer than 10 bp were merged according to their overlap with mismatch no more than 2 bp, (iii) Sequences of each sample were separated according to the barcodes (exactly matching), and (iv) Primers (allowing two nucleotide mismatching) and reads containing ambiguous bases were removed. Operational taxonomic units (OTUs) were clustered with 97% similarity cutoff by using the UPARSE (version 7.1)^1^ with a novel ‘‘greedy’’ algorithm that performs the chimera filtering and OTU clustering simultaneously. The taxonomy of each *16S rRNA* gene sequence was analyzed by the ribosomal database project (RDP) Classifier Algorithm^2^ against the Silva (SSU123) 16S rRNA database by using a confidence threshold of 70%.

The α-diversity of gut microbiota was described separately by the Ace index and the Shannon index. The β-diversity of gut microbiota was analyzed by the principal coordinate analysis (PCoA) and hierarchical clustering analysis (HCA). The compositional similarity of microbiota in the two groups was reflected by the Venn analysis at the OTU level. The difference in microbial composition between the groups was more intuitive and visualized through the community Bar plot analysis. The significant differences in the phylum and genus levels were tested according to the relative abundance of the two groups of samples. The correlation values of the gut microbiota and inflammatory factors in serum are visually evaluated by using the correlation heatmap graphs.

### Untargeted Metabolomics Analysis

Fecal samples (50 mg) were suspended in a 400 μl extraction solution (methanol: water = 4:1), crushed at −20°C with the high-throughput tissue disrupter (60 Hz), vortexed, and extracted by ultrasonic on ice for 10 min three times (40 Hz, 300 W). After standing at −20°C for 30 min, the extracted samples were centrifuged at 13,000 *g* for 15 min at 4°C, removed the supernatant, and transferred to a 200 L vial for UPLC-Q-TOF/MS analysis. The pooled quality control (QC) sample was prepared by mixing equal volumes of all the samples. The QC samples were disposed of and tested in the same manner as the analytic samples, which would be injected every six samples in order to monitor the stability of the analysis. Chromatographic separation of the metabolites was performed on the ExionLC AD System (AB SCIEX, United States) equipped with the ACQUITY UPLC BEH C18 Column (100 mm × 2.1 mm in diameter, 1.7 μm; Waters, Milford, MA, United States). The mobile phases consisted of 0.1% formic acid in water with formic acid (0.1%) (solvent A) and 0.1% formic acid in acetonitrile:isopropanol (1:1, v/v) (solvent B). The solvent gradient changed according to the following conditions: from 0 to 3 min, 95% (A): 5% (B) to 80% (A): 20% (B); from 3 to 9 min, 80% (A): 20% (B) to 5% (A): 95% (B); from 9 to 13 min, 5% (A): 95% (B) to 5% (A): 95% (B); from 13 to 13.1 min, 5% (A): 95% (B) to 95% (A): 5% (B); from 13.1 to 16 min, 95% (A): 5% (B) to 95% (A): 5% (B) for equilibrating the systems. The sample injection volume was 20 μl and the flow rate was set to 0.4 ml/min. The column temperature was maintained at 40°C. During the period of analysis, all these samples were stored at 4°C. The UPLC system was coupled to a quadrupole-time-of-flight mass spectrometer (Triple TOFTM 5600+, AB SCIEX, United States) equipped with an electrospray ionization (ESI) source operating in the positive mode and negative mode. The optimal conditions were set as follows: source temperature, 500°C; curtain gas (CUR), 30 psi; both ion source GS1 and GS2, 50 psi; ion spray voltage floating (ISVF), −4,000 V in the negative mode and 5,000 V in positive mode, respectively; declustering potential, 80 V; collision energy (CE), 20–60 V rolling for MS/MS. Data acquisition was performed with the data-dependent acquisition (DDA) mode. The detection was carried out over a mass range of 50–1,000 m/z. The detailed methods were provided in the Supplementary Methods. Differential metabolites analysis, the Kyoto Encyclopedia of Genes and Genomes (KEGG) enrichment analysis, and correlation analysis with the bacterial flora and differential metabolites were completed on the free online Majorbio I-Sanger Cloud Platform.^3^ The differential metabolites between the groups were analyzed by the orthogonal partial least squares discriminant analysis (OPLS-DA) method [variable importance in projection (VIP) > 1, *p* < 0.05]. The fold changes of the metabolite ratio of the model group (numerator) to the control group (denominator) and the expression levels of differential metabolites between the normal group and the model group are listed in the Supplementary Materials.

### Statistical Analysis

Data are expressed as the mean ± SEM. The analysis methods of cytokine data were the Mann–Whitney test for interleukin-1β (IL-1β), interleukin-6 (IL-6), and tumor necrosis factor-α (TNF-α) and the unpaired *t*-test for interleukin-4 (IL-4), interleukin-10 (IL-10), interferon-γ (IFN-γ), macrophage colony-stimulating factor (M-CSF), and chemokine (C-X-C motif) ligand 1 (CXCL1). The analysis of significant differences between groups in the phylum and genus levels was tested by the Wilcoxon rank-sum test. The significance of differences between the two groups was evaluated for ^∗^*p* < 0.05, ^∗∗^*p* < 0.01, and ^∗∗∗^*p* < 0.001.

## Results

### Histopathological Observation of Gastric Mucosa and Detection of Serum Inflammatory Cytokines

**FIGURE 2:**
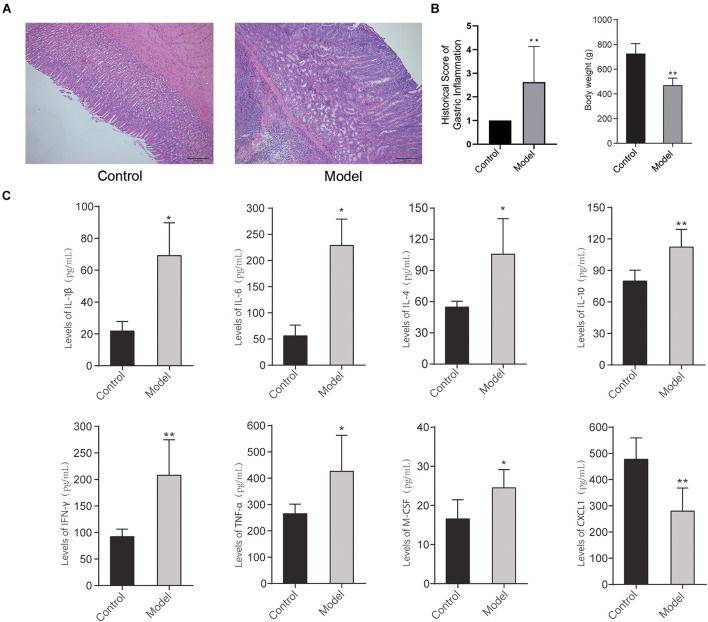
**(A)** Histopathological image and inflammation score of the gastric mucosa of the control group and model group stained with H&E dying. **(B)** Bodyweight of rats in the control group and model group at 32 weeks. **(C)** The levels of inflammatory cytokines in the serum of the rats in the control group and model group (*p < 0.05, **p < 0.01). *n* = 9.

The histopathological images of the gastric mucosa of the control group and model group are shown in Figure 2A. The results showed that with respect to the control group, there was obvious gastric inflammation in the rats of the model group, there was a significant polymorphonuclear cells infiltration in the mucosa, submucosa, and muscularis. In addition, the bodyweight of rats at 32 weeks decreased significantly compared with the control group (Figure 2B). It indicated that multifactor induction could cause precancerous lesions of rat gastric mucosa, which was consistent with our previous report (Yu et al., 2020).

To further detect the systematic inflammatory state of the rats, the levels of serum inflammatory cytokines were detected by the Bio-Plex Pro Rat Cytokine 23-Plex Group I Panel System (Bio-Plex). As shown in Figure 2C, after multifactor stimulation, the levels of inflammatory cytokines in the serum in the model group were significantly different from the control group. Among them, the levels of IL-1β, IL-4, IL-6, IL-10, IFN-γ, TNF-α, and M-CSF in serum of the rats in the model group were significantly increased, while the level of CXCL1 in serum of the rats in the model group was significantly reduced compared with the control group. It indicated that the differential role of CXCL1 takes place in the formation of PLGC.

### Alteration in the Composition and Function of Gut Microbiota After Multifactor Induction

**FIGURE 3:**
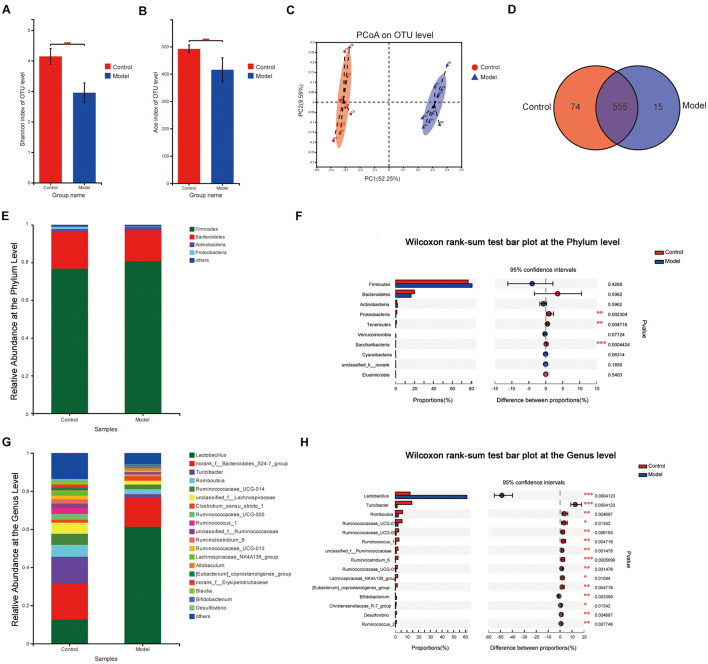
Microbiota change after multifactor stimulation. **(A)** The Shannon index. **(B)** The Ace index. **(C)** Principal coordinate analysis (PCoA) analysis of β-diversity. **(D)** Venn analysis of gut microbiota. **(E,G)** The relative abundance at the phylum and genus levels. **(F,H)** Statistical results of the Wilcoxon rank-sum test in the phylum and genus levels (*p < 0.05, **p < 0.01, ***p < 0.001). *n* = 9.

In total, 8,99,441 valid *16S rRNA* gene sequences were obtained from 18 rat stool samples. The analysis was performed at a level of similarity higher than 97% and a total of 644 OTUs were obtained. According to the Shannon index (control vs. model: 4.144 ± 0.256 vs. 2.954 ± 0.321, *p* < 0.001) and the Ace index (control vs. model: 492.01 ± 14.50 vs. 415.51 ± 44.01, *p* < 0.001), there was a significant difference in microbiota diversity and richness between the two groups. It indicated that the Shannon index and the Ace index of the microbiota in the model group reduced significantly compared with the control group (Figures 3A,B). As shown in Figure 3C, the β-diversity analyzed by PCoA indicated that there was a significant difference between the two groups on the OTU level and good similarity within the group. The Venn analysis showed that the total number of microbiotas in both groups was 555 and the number of unique microbiotas in the control group was 74, while that in the model group was 15 (Figure 3D).

The microbial compositions between the groups were significantly different on the phylum and genus levels (Figures 3E,G). The results revealed that the predominant phyla in both the groups were Firmicutes (76.83% in the control group and 80.85% in the model group) and Bacteroidetes (19.81% in the control group and 16.31% in the model group) on the phylum level, while there was significantly reduced proportion of *Proteobacteria*, *Tenericutes*, and *Saccharibacteria* in the model group compared with the control group. On the genus level, 67 genera were significantly different (*p* ≤ 0.05) between the two groups. Compared to those in the control group, *Lactobacillus* and *Bifidobacterium*, two major probiotics with anti-inflammatory activity, increased significantly, while *Turicibacter*, *Romboutsia*, *Ruminococcaceae_UCG-014*, *Ruminococcaceae_UCG-005*, and *Ruminococcus_1* reduced significantly in the model group (Figures 3F,H).

### Inflammatory Cytokines Increasing Are Correlated With Differential Bacteria

**FIGURE 4:**
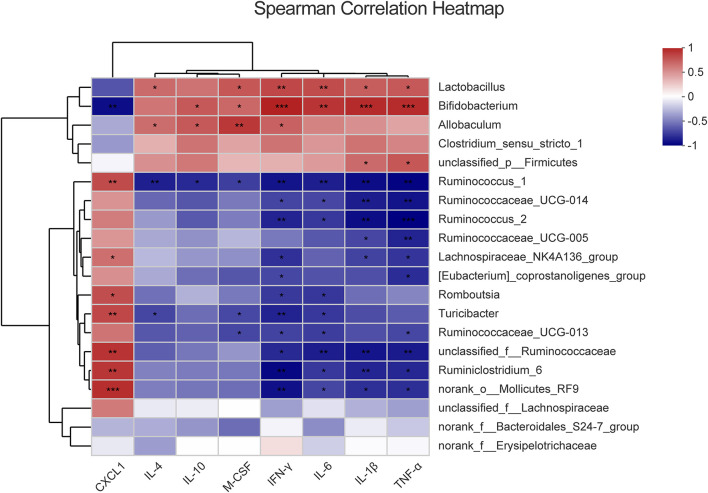
The correlation between the different cytokines and differential intestinal microbes, respectively. The color represents the correlation coefficient. *p < 0.05, **p < 0.01, ***p < 0.001.

The imbalance of the intestinal flora is associated with the inflammatory response of the host. To assess the correlation of the differential systemic inflammation and microbiota changes between the control group and model group, Spearman’s rank correlation coefficient was used for the differential microbiota (*p* < 0.05) and the inflammatory cytokines. The heatmap is ordered by the correlation level and significance (*p*-value) within the top 20 floras shown in Figure 4 and 17 floras are significantly correlated with the serum cytokines level alternation. Among them, there was a significant positive correlation between IL-4, IL-10, M-CSF, IFN-γ, IL-1β, TNF-α, and IL-6 with *Bifidobacterium*, *Lactobacillus*, *Allobaculum* and a negative correlation with *Ruminococcus_1*, *Romboutsia*, *Ruminococcaceae*, *Ruminiclostridium_6*, and *Turicibacter*. On the contrary, CXCL1 showed a converse trend of correlation compared with other cytokines, which mainly attributed to the opposite change profile of CXCL1 in the model group (Figure 2C). In addition, the *Ruminococcaceae* family, *Bifidobacterium*, and *Lactobacillus* are most relative to cytokines change among all the differential flora at the genus level. It indicated that intestinal floras might regulate the inflammatory cytokines in the serum of model rats and the *Ruminococcaceae* family, *Bifidobacterium*, and *Lactobacillus* are most potentially involved in the intestinal homeostasis disorder and PLGC formation.

### Analysis of Differential Metabolites in the Stool Samples

**FIGURE 5:**
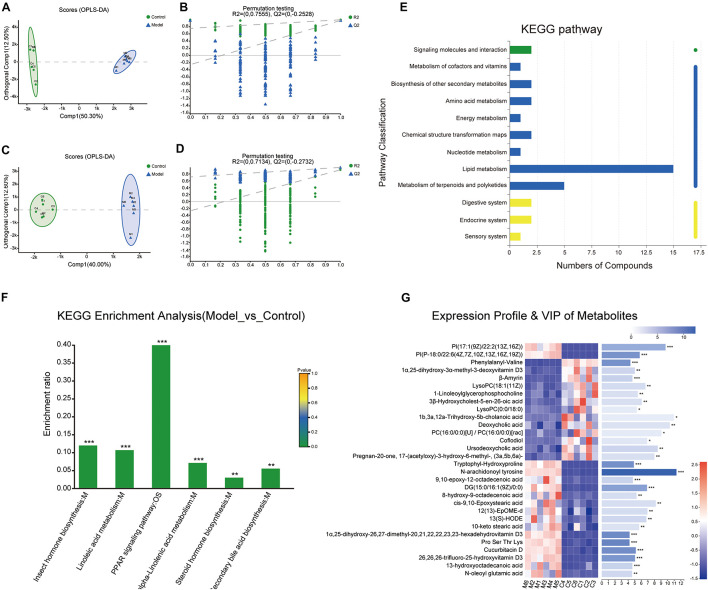
Metabolites analysis between the control and model groups. **(A–D)** Orthogonal partial least squares discriminant analysis (OPLS-DA) score plots of fecal metabolites in comparisons of the control group and model group in the positive and negative ion modes. **(E)** The Kyoto Encyclopedia of Genes and Genomes (KEGG) functional pathway analysis. The abscissa is the number of metabolites annotated to this pathway and the ordinate is the name of the KEGG metabolic pathway. **(F)** KEGG enrichment analysis. **(G)** Expression profile and variable importance in projection (VIP) of the top 30 differential metabolites analyzed through heatmap analysis methods. (***p < 0.001, **p < 0.01, *p < 0.05). *n* = 6.

To further explore the interaction mechanism between microbiota and the systematic inflammatory response, total ion chromatograms of the stool sample were detected by using UPLC-Q-TOF/MS in the positive and negative ion modes. Samples were analyzed by the OPLS-DA and the OPLS-DA score plots presented a distinct clustering of metabolites in the stool samples between the control group and model group in both the positive (Figures 5A,B) and negative (Figures 5C,D) ion modes, which suggested that the fecal metabolic profiles had been changed after intervened by the multifactor stimulation.

Next, a total of 337 significantly differentially expressed metabolites with annotated names were detected under the screening conditions of *p* < 0.05 and VIP > 1 (Supplementary Table 1). The expression profile and VIP of the top 30 differential metabolites such as N-arachidonoyl tyrosine, Pro-Ser-Thr-Lys, cucurbitacin D, 1α,25-dihydroxy-26,27-dimethyl-17,20,21,22,22,23,23-hexadehydrovitamin D3, tryptophylhydroxyproline, and phenylalanylvaline were showed as a heatmap in Figure 5G and the full chemical information of the metabolites of the top 30 differential metabolites was shown in Table 1. Besides, the KEGG functional pathway and enrichment analysis of the differential metabolites found that the differential metabolites are mainly related to metabolism, peroxisome proliferator-activated receptor (PPAR) signaling pathway, insect hormone biosynthesis, (alpha-) linoleic acid metabolism, steroid biosynthesis, secondary bile acid biosynthesis, and steroid hormone biosynthesis (Figures 5E,F).

**TABLE 1:** The chemical information of the top 30 differential metabolites.

Metabolite	Mode	M/Z	RT (min)	VIP value	P value	Fold changes (Control/Model)	HMDB Subclass
N-arachidonoyl tyrosine	pos	468.306	3.9072	11.3507	5.982E-13	6.1857	Amino acids, peptides, and analogs
Pro Ser Thr Lys	pos	432.2408	4.6296	4.2109	1.139E-09	3.8443	Amino acids, peptides, and analogs
1α,25-dihydroxy-26,27-dimethyl-20,21,22,22,23,23-hexadehydrovitamin D3	pos	439.3152	4.3854	4.2169	3.083E-09	3.3114	Vitamin D and derivatives
Cucurbitacin D	pos	517.3244	4.8337	5.1645	3.993E-09	2.6176	Cucurbitacins
Tryptophyl-Hydroxyproline	pos	300.1373	1.2872	4.8653	5.607E-09	14.2487	Amino acids, peptides, and analogs
Phenylalanyl-Valine	pos	297.1799	2.1592	4.3459	7.864E-09	0.1724	Amino acids, peptides, and analogs
PI[P-18:0/22:6(4Z, 7Z, 10Z, 13Z, 16Z, 19Z)]	pos	895.5791	8.5257	5.7623	1.241E-08	119.4175	Amino acids, peptides, and analogs
26,26,26-Trifluoro-25-hydroxyvitamin D3	pos	455.3099	4.2291	5.0392	1.924E-08	3.5673	Vitamin D and derivatives
DG[15:0/16:1(9Z)/0:0]	pos	575.4659	9.4817	6.8613	1.057E-07	6.8347	Diradylglycerols
PI[17:1(9Z)/22:2(13Z,16Z)]	pos	903.5846	8.3	9.6808	7.928E-07	2976.4356	Glycerophosphoinositols
β-Amyrin	pos	409.3816	10.2151	4.6561	0.000575	0.1848	Triterpenoids
13-hydroxyoctadecanoic acid	pos	283.262	7.8558	4.642	0.0006128	4.395	Fatty acids and conjugates
9,10-epoxy-12-octadecenoic acid	pos	297.2413	6.4444	4.7207	0.0008167	6.3232	Lineolic acids and derivatives
1α,25-dihydroxy-3α-methyl-3-deoxyvitamin D3	pos	415.3556	9.3034	5.0118	0.001137	0.2422	Vitamin D and derivatives
Ursodeoxycholic acid	pos	357.2779	7.0011	8.5	0.001209	0.6283	Bile acids, alcohols and derivatives
13(S)-HODE	pos	297.2413	7.6725	6.7751	0.001627	3.2121	Lineolic acids and derivatives
N-oleoyl glutamic acid	pos	412.3041	4.9695	4.5913	0.001791	4.2917	Amino acids, peptides, and analogs
LysoPC[18:1(11Z)]	pos	522.3538	8.3284	6.6158	0.003252	0.3125	Glycerophosphocholines
8-hydroxy-9-octadecenoic acid	pos	299.2571	7.8453	5.2733	0.004067	8.2188	Lineolic acids and derivatives
3β-Hydroxycholest-5-en-26-oic acid	pos	480.3438	8.4665	6.0719	0.005779	0.1319	Bile acids, alcohols and derivatives
(3a,5b,6a)-3-hydroxy-6-methyl-17-(acetyloxy)-Pregnan-20-one	pos	391.2833	6.2504	7.9849	0.005887	0.3124	Pregnane steroids
1-Linoleoylglycerophosphocholine	pos	520.3382	7.8047	5.4651	0.006243	0.4019	Glycerophosphocholines
Coflodiol	pos	425.3763	9.2733	6.8958	0.01892	0.1551	Triterpenoids
PC(16:0/0:0)[U]/PC(16:0/0:0)[rac]	pos	496.3384	8.2299	9.0627	0.03449	0.4909	Glycerophosphocholines
LysoPC(0:0/18:0)	pos	524.3696	8.8922	5.3144	0.03774	0.451	Glycerophosphocholines
1b,3a,12a-Trihydroxy-5b-cholanoic acid	pos	817.5801	5.3651	10.9706	0.04306	0.0896	Bile acids, alcohols, and derivatives

### Metabolites Are Correlated With Intestinal Differential Microbes

**FIGURE 6:**
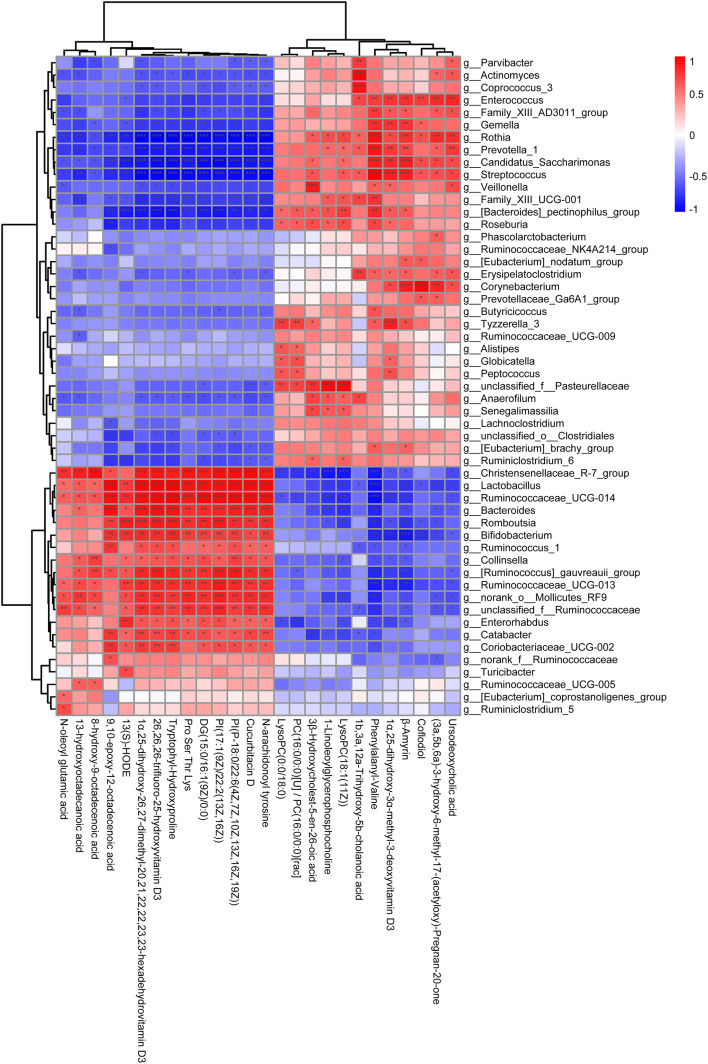
The correlation analysis of the differential metabolites and intestinal microbes in the genus level. The ordinate represents the differential metabolites and the abscissa represents the intestinal microbes in the genus level. The color represents the correlation coefficient (***p < 0.001, **p < 0.01, *p < 0.05).

To further explore the relationship between the intestinal microbial changes and differences in metabolites, the correlation between the differential metabolites (*p* < 0.05) and intestinal microbes was analyzed by correlation heatmap analysis to calculate the Spearman’s rank correlation coefficient. As shown in Figure 6, there is a good clustering relationship in the differential metabolites and intestinal microbes, respectively, which may indicate that they have similarities in structural and biological function. The *Bifidobacterium* and *Lactobacillus*, which is the main genus related to the alternation of the cytokines (Figure 4), shared the same correlation profile of metabolic (Figure 5). On the contrary, the genus within the *Ruminococcaceae* family does not have the same correlation profile. Among all the metabolic, N-arachidonoyl tyrosine, cucurbitacin D, PI [P-18:0/22:6(4Z,7Z,10Z,13Z,16Z,19Z)], Phosphatidyl inositols (PI) [17:1(9Z)/22:2(13Z,16Z)], diacyl glycerol (DG) [15:0/16:1(9Z)/0:0], Pro-Ser-Thr-Lys, tryptophylhydroxyproline, 26,26,26-trifluoro-25-hydroxyvitamin D3, 1α,25-dihydroxy-26,27-dimethyl-17,20,21,22,22,23,23-hexadehydrovitamin D3, 13(S)-HODE, and 10-epoxy-12-octadecenoic acid were mainly positively associated with the genus *Lactobacillus* and *Bifidobacterium* and ursodeoxycholic acid, β-amyrin, phenylalanylvaline, and 1α,25-dihydroxy-3α-methyl-3-deoxyvitamin D3 were mainly negatively associated with the genus *Lactobacillus* and *Bifidobacterium*.

## Discussion

Gastric cancer is an important health problem with the fifth most common cancer and the third leading cause of cancer-related death worldwide. There are about 1.2 million new cases of GC worldwide each year and about 40% of them occur in China. The risk factors of GC development have been identified to some extent such as *H. pylori* infection, dietary habits, nitrite intake, and PPI abuse. But, some of the mechanisms between risk factors remain unclear. Gut microbial dysbiosis are linked to the aberrant immune responses accompanied by abnormal production of inflammatory cytokines. Validation of the predicted host–microbial interactions reveals that TNF-α and IFN-γ production are associated with specific microbial metabolic pathways such as palmitoleic acid metabolism and tryptophan degradation to tryptophol (Schirmer et al., 2016). This study aims to explore how multirisk factors influence the gut microbiota and gastric inflammation and the underlying mechanisms. We reported that the gut microbial dysbiosis and changes in fecal metabolic phenotype in PLGC rats could stimulate the immune responses to regulate the levels of a variety of inflammatory cytokines, thereby accelerating the formation of PLGC.

The malignant transformation of chronic inflammation is a common process in the development of most cancers. The persistence of chronic inflammation plays an important role in initiating, maintaining, and promoting the growth of GC. The possibility of abnormal expansion of cancer cell DNA was promoted by increasing the infiltration of chemokines on the gastric mucosal epithelial cells. Inflammatory cytokines such as IL-1β, IL-6, and TNF-α are often used to assess the severity of gastric mucosal injury (Michalkiewicz et al., 2015). IL-1β is a typical proinflammatory cytokine. Inflammatory signals can activate the specific immune responses by activating inflammatory corpuscles IL-1β, stimulating secreted IL-1β with a small amount of expression to produce the appropriate inflammatory responses (Apte and Voronov, 2002). A large number of IL-1β can cause a wide range of inflammatory reactions, leading to inflammatory damage. IL-6 is regarded as a proinflammatory cytokine that can participate in the tumor development and development process through the mechanisms of promoting tumor vascular generation, regulating the genes related to the cell cycle, accelerating the speed of tumor stem cell occurrence and self-renewal, and regulating the local inflammatory environment of the body. Gastric mucosal inflammatory lesions lead to an abnormal rise in IL-6 serum cytokines, which have important reference value in detecting the inflammation and pathological degree (Hodge et al., 2005; Nilsson et al., 2005; Landskron et al., 2014). IL-4 can mediate the activation of T cells and B cells and cause excessive immune inflammatory injury in tissues (Fang et al., 2020). The cytokine IL-10 is a key anti-inflammatory cytokine to protect the host from overexuberant responses to microbiota and pathogens and functions as a negative regulator of immune responses to microbial antigens while playing important roles in maintaining the intestinal microbe–immune homeostasis, sterile wound healing, autoimmunity, cancer, and homeostasis (Neumann et al., 2019; Saraiva et al., 2020). IFN-γ is the uppermost cytokine implicated in antitumor immunity with cytostatic, proapoptotic, and immune-provoking effects. Many clinical trials and immunotherapy approaches have been designed to reinforce IFN-γ-mediated immunity for the different types of cancer (Kursunel and Esendagli, 2016). TNF-α is a typical proinflammatory factor produced during the initiation of inflammation to maintain chronic inflammation, promote the expression of other inflammatory cytokines, aggravate inflammation, and play an important role in the inflammation and tumor genesis. It had been reported that the level of TNF-α increased in *H. pylori*-positive PLGC, through *H. pylori* secreted TNF-α-inducing protein (Tipα) (Suganuma et al., 2012; Landskron et al., 2014). M-CSF is related to monocytes and macrophages proliferation. Elevated M-CSF is correlated with invasion, metastasis, and poor survival of patients with tumors. M-CSF, combined with IL-34, tumor associated macrophages (TAMs) were the novel biological markers for GC, which may provide new insight for both the diagnosis and cellular therapy of GC (Liu et al., 2020). CXCL1 can promote and enhance the killing of macrophages to tumor cells and microorganisms, regulate the release of cytokines and other inflammatory regulators by macrophages, and stimulate cell phagocytosis. CXCL1, one of the CXCR2 ligands secreted by macrophages in the GC microenvironment, could promote migration of GC cells through activating the CXCR2/STAT3 feed-forward loop. TNF-α secreted by GC cells could induce the release of CXCL1 by macrophages (Zhou et al., 2019). Recently, with the constitutional understanding of the tumor microenvironment, the role of several cytokines needs to be revalued. The anti-inflammatory cytokines such as IL-4 and IL-10, which were considered protected cytokines in the past, become a double-edged sword in the protection of cancer. On one side, anti-inflammatory cytokines are important to alleviate inflammation, a huge risk factor of cancerization. On the other side, most of the anti-inflammatory cytokines could suppress the immune response, which is important for mutation surveillance and elimination (Salazar-Onfray et al., 2007; Li et al., 2009; Musolino et al., 2017). This study showed that both the proinflammatory and anti-inflammatory cytokines are increased in the model group, except CXCL1, which indicated the systematic activation of the immune system. The CXCL1 is often cosecreted with inflammatory cytokines such as IL-1, IL-6, and TNF-α by infiltrating macrophage or cancer cells. The decrease of CXCL1 of the model group described that the upstream of multifactor-induced systematic immune activation is different from the normal activation pathway. The multifactor induction has a wide effect on both the digestion system and other organs in rats, the underlying mechanism of it still needed further study.

In addition, we try to link systematic inflammation with intestinal microbiota alternation. This study showed that *Lactobacillus* and *Bifidobacterium* are increased significantly in the model group, which makes us reconsider their role in cancer. In this study, unlike in other diseases, the *Lactobacillus* and *Bifidobacterium* are potentially contributed to the development of cancer (Dicksved et al., 2009; Aviles-Jimenez et al., 2014; Castano-Rodriguez et al., 2017). Some researchers attribute the abnormal role of *Bifidobacterium* and *Lactobacillus* in cancer to the unusual cancer metabolic environment (Vinasco et al., 2019). This study revealed a strong positive correlation between *Lactobacillus* and *Bifidobacterium* with serum cytokines arising in PLGC rats and some of the flora shared a similar clustering profile of metabolic including *Bifidobacterium* and *Lactobacillus*. All of these pointed out the vital role of *Lactobacillus* and *Bifidobacterium* in precancerous lesions and the potential to study the metabolic environment of these two floras.

The microbiota is associated with host systemic metabolites, most of the metabolic released by floras are shared across the subjects (Visconti et al., 2019). In our healthy GI tract, there are over 90% of the bacteria belong to the phyla of *Firmicutes* and *Bacteroidetes*, followed by *Actinobacteria*, *Proteobacteria*, *Verrucomicrobia*, and *Fusobacteria*, which are in a relatively stable balance (Bäckhed et al., 2005; Qin et al., 2010). Gut microbiota could produce several metabolites and bioproducts necessary to protect the health and gastrointestinal homeostasis of the host. However, the constitution and metabolic function of gut microbiota are easily influenced by a variety of factors including genetics, diet, age, antibiotics, mode of delivery, stress, and environmental and psychological factors (Osadchiy et al., 2019). The disorders in the constitution and metabolic function of gut microbiota could be increasingly linked to various diseases including inflammation, cancers, obesity, metabolic diseases, allergies, depression, and disorders in the immune system (Turnbaugh et al., 2007; Shanahan, 2013). The increasing evidence has confirmed that the disturbance of the composition and function of the GI tract microorganism is closely related to the occurrence of GI inflammation and cancers, likely due to altered mucosal immunity and proinflammatory immune microenvironment (Aviles-Jimenez et al., 2014; Brawner et al., 2014; Coker et al., 2018; Thakkar et al., 2018; Kong and Cai, 2019). Ranitidine, a potent histamine H2 receptor antagonist inhibiting gastric acid secretion, could cause an imbalance of gut microbiota, including the excessive proliferation of *H. pylori* and other microorganisms in the GI tract (Zeldis et al., 1983).

In addition to the gut microbiota involved in human health and disease, microbial metabolites or cometabolites from both the host and microbes can contribute to inflammatory and regulation of the immune, endocrine, and nervous system. The disorder of gut microbes also leads to the production of harmful metabolites such as acetaldehyde, secondary bile acids, and glucuronic acid to induce DNA damage and contribute to carcinogenesis (Kong and Cai, 2019). We next proved that the metabolic change of microbiota is highly associated with host metabolism homeostasis. On this basis, we exerted the metabolomics methods to bridge the gut microbiota with precancerous inflammation. The results showed that the control group and model group are significantly different, especially on the lipid metabolism. It also partly revealed the potential mechanism of how alternation of gut microbiota contributes to the PLGC formation. The molecule correlated with differential microbiota deserves future exploration and more convincing evidence is still needed.

This study provides a comprehensive view of gut microbiota, inflammation, and gut metabolism in the PLGC stage in the rat. Besides, we brought new evidence of the precancerous role of *Lactobacillus* and *Bifidobacterium*, which is of interest to clinical microbiologists and physicians. However, the crosstalk of specific floras, metabolic, and cytokines needs further exploration and validation.

## Data Availability Statement

The datasets presented in this study can be found in online repositories. The names of the repository/repositories and accession number(s) can be found below: https://www.ncbi.nlm.nih.gov/Traces/study/?acc=PRJNA752833.

## Ethics Statement

The animal study was reviewed and approved by Animal Ethics Committee of Beijing University of Chinese Medicine, China (No. BUCM-4-2017120101-4038).

## Author Contributions

FC, ZS, and XD conceived and designed the experiments. YuL, HZ, XM, and TL analyzed and interpreted the data. YiL, MZ, JL, and TX carried out animal experiments. PZ carried out a pathology experiments and analysis. FC wrote the manuscript. All the authors read and approved the final manuscript.

## Conflict of Interest

The authors declare that the research was conducted in the absence of any commercial or financial relationships that could be construed as a potential conflict of interest.

## Publisher’s Note

All claims expressed in this article are solely those of the authors and do not necessarily represent those of their affiliated organizations, or those of the publisher, the editors and the reviewers. Any product that may be evaluated in this article, or claim that may be made by its manufacturer, is not guaranteed or endorsed by the publisher.

## Footnotes

1
http://drive5.com/uparse/

2
https://sourceforge.net/projects/rdp-classifier/

3
www.i-sanger.com

## Supplementary Material

Supplementary Table 1 The significantly differential expressed metabolites in the stool samples of the control group and model group rats.
